# Characterising antibody avidity in individuals of varied *Mycobacterium tuberculosis* infection status using surface plasmon resonance

**DOI:** 10.1371/journal.pone.0205102

**Published:** 2018-10-12

**Authors:** Simon G. Kimuda, Irene Andia Biraro, Bernard S. Bagaya, John G. Raynes, Stephen Cose

**Affiliations:** 1 Department of Medical Microbiology, School of Biomedical Sciences, Makerere University College of Health Sciences, Kampala, Uganda; 2 Immunomodulation and Vaccines Programme, Medical Research Council/ Uganda Virus Research Institute and London School of Hygiene & Tropical Medicine Uganda Research Unit, Entebbe, Uganda; 3 Department of Internal Medicine, School of Medicine, Makerere University College of Health Sciences, Kampala, Uganda; 4 Department of Immunology and Molecular Biology, School of Biomedical Sciences, Makerere University College of Health Sciences, Kampala, Uganda; 5 London School of Hygiene & Tropical Medicine, London, United Kingdom; University of Cape Town, SOUTH AFRICA

## Abstract

There is increasing evidence supporting a role for antibodies in protection against tuberculosis (TB), with functional antibodies being described in the latent state of TB infection. Antibody avidity is an important determinant of antibody-mediated protection. This study characterised the avidity of antibodies against Ag85A, an immunodominant *Mycobacterium tuberculosis* (*M*.*tb*) antigen and constituent of several anti-TB vaccine candidates, in individuals of varied *M*.*tb* infection status. Avidity of Ag85A specific antibodies was measured in 30 uninfected controls, 34 individuals with latent TB infection (LTBI) and 75 active pulmonary TB (APTB) cases, employing the more commonly used chaotrope-based dissociation assays, and surface plasmon resonance (SPR). Chaotrope-based assays indicated that APTB was associated with a higher antibody avidity index compared to uninfected controls [adjusted geometric mean ratio (GMR): 1.641, 95% confidence interval (CI): 1.153, 2.337, p = 0.006, *q* = 0.018] and to individuals with LTBI [adjusted GMR: 1.604, 95% CI: 1.282, 2.006, p < 0.001, *q* <0.001]. SPR assays showed that APTB was associated with slower dissociation rates, an indication of higher avidity, compared to uninfected controls (adjusted GMR: 0.796, 95% CI: 0.681, 0.932, p = 0.004, *q* = 0.012) and there was also weak evidence of more avid antibodies in the LTBI compared to the uninfected controls (adjusted GMR: 0.871, 95% CI: 0.763, 0.994, p = 0.041, *q* = 0.123). We found no statistically significant differences in anti-Ag85A antibody avidity between the APTB and LTBI groups. This study shows that antibodies of increased avidity are generated against a principle vaccine antigen in *M*.*tb* infected individuals. It would be important to determine whether TB vaccines are able to elicit a similar response. Additionally, more research is needed to determine whether antibody avidity is important in protection against infection and disease.

## Introduction

According to the WHO, tuberculosis (TB) was the leading cause of mortality from a single infectious microorganism in 2016 [1]. This illness is caused by the pathogen, *Mycobacterium tuberculosis* (*M*.*tb*) and most commonly affects the lungs. Although treatment exists, there are increasing rates of resistance towards the principal drugs used in anti-mycobacterial chemotherapy [2]. The vaccine, *Mycobacterium bovis* Bacille Calmette–Guérin (BCG) protects children from acquiring *M*.*tb* infection [3,4] and from developing severe forms of TB disease such as TB meningitis and military TB [5]. Despite extensive research, the exact mechanisms of BCG induced protection are still not known [6], although the presence of interferon (IFN)-γ secreting T cells has been associated with a lower risk of TB in a study of BCG vaccinated infants [7]. This same study also reported elevated levels of antibodies against the immunodominant secreted protein, Ag85A, in children who did not develop TB.

Studies of immunological responses in *M*.*tb* infection over the years have pointed to the contribution of CD4+ T cells and the cytokines they produce, IFN-γ and TNF-α. These immune cells and cytokines promote local containment of mycobacteria [8,9]. There has been less emphasis on the study of antibody immune responses against *M*.*tb*, partly due to results of early studies involving transfer of immune sera to TB cases that showed inconsistent efficacy, were difficult to reproduce and lacked appropriate controls [10,11]. Studies in murine models conducted since then have explored the impact of the absence of B cells on mycobacterial control *in vivo* and have also yielded contradictory results. Johnson *et al*. [12] showed that the absence of antibodies did not impact on the course of mycobacterial disease while studies by Vordermeier *et al*. [13] and later by Torrado *et al*. [14] indicated that the absence of B cells and/or antibodies resulted in higher mycobacterial burden in the lungs. Studies in human participants have shown that anti-mycobacterial antibodies promote phagocytosis of mycobacteria and subsequent killing of engulfed bacilli in *in vitro* models [15] in support of an active role of antibodies in the immune response against mycobacterial infections. These antibodies also activate complement, leading to the destruction of mycobacterial cells and release of molecules that promote inflammation [16,17]. Antibodies enhance intracellular destruction of bacilli via antibody-dependent cell cytotoxicity [18] and antibodies of the IgA isotype have been shown to prevent infection of human lung epithelial cells but curiously not those of the IgG class, the most abundant [19]. Antibody responses are usually higher in active TB disease compared to LTBI [20–22], a state in which mycobacteria are mostly controlled. The common observation from serological studies is that antibody titres increase with severity of disease and are associated with mycobacterial load [23,24]. The presence of antibodies against *M*.*tb* proteins such as alanine dehydrogenase has even been associated with treatment failure [25]. This implies that anti-*M*.*tb* antibodies may not be protective in active disease.

A study by Lu and colleagues has shown that despite high antibody titres in active TB disease, antibodies in this infection state are less able to elicit functional responses compared to those found in LTBI [18]. The authors attributed the reduction in antibody functionality to alterations in antibody Fc regions in active TB that may influence interactions with Fc receptors on immune cells. However, the study did not investigate the affinity of antibody Fab regions for their cognate antigens yet this property is an important determinant of antibody-mediated protection [10]. Antibody affinity may influence the strength of interaction between antibodies and their respective antigens and may consequently affect their ability to neutralise microbial toxins or prevent infection of cells [26]. A study by Perley and colleagues demonstrated that antibodies in active TB cases compared to uninfected controls had reduced binding avidity for the surface of live mycobacteria [27]. However, it also reported higher antibody avidity for mycobacterial whole cell lysates and secreted proteins. The measure of avidity presented in the Perley study was based on the use of chaotropic reagents to disrupt antibody-antigen binding. Although this provides a proxy measure of avidity or functional affinity [28], it does not capture information on the rate of dissociation of an antibody from its cognate antigen, which is a more accurate measure of how long antibodies are able to remain bound to microbial antigens and mediate effector functions [29]. Indeed, antibody Fc-Fc receptor signalling strength, a determinant of antibody-mediated effector function, has been shown to be associated with antibody dissociation rate [30]. Antibody-antigen interactions can now be assayed in real time by surface plasmon resonance (SPR) technology, providing a measure of dissociation rate. This study characterised the avidity of antibodies against Ag85A, an immunodominant *M*.*tb* antigen and constituent of several new anti-TB candidate vaccines [31], in uninfected controls, individuals with LTBI and those with active pulmonary TB (APTB) from a TB household contact study using surface plasmon resonance. We compared the findings from this method to those from the more commonly used chaotrope-based dissociation assays.

## Materials and methods

### Study population and design

This study was of a cross-sectional design and was nested within a research study in Kampala, Uganda, investigating the influence of parasitic and viral infections among household contacts (HHCs) of APTB cases on their susceptibility to *M*.*tb* infection [32]. The parent study recruited 101 individuals with APTB living in Kisenyi and Kitebi; two suburbs of Kampala city and then recruited 291 of their HHCs. The APTB cases had to be over 18 years and had either just begun anti-mycobacterial drug therapy or had been on treatment for not more than 4 weeks. The individuals recruited as HHCs had to have shared a home or meals with the APTB cases for at least 2 weeks before their diagnosis. APTB was defined by sputum positivity for acid-fast bacilli. Among the HHCs, the main outcome, LTBI was defined by positive tuberculin skin test (TST) and positive QuantiFERON-TB Gold in-tube (QFT-GIT) test in the absence of signs or symptoms of APTB. The HHCs were considered to be uninfected if they had a negative TST and negative QFT-GIT test result. The participants included in our study were 139 in total and comprised 75 APTB cases and from among the HHCs; 34 individuals with LTBI and 30 uninfected controls. The study was exploratory and the sample size was determined by the availability of sufficient sample volumes for laboratory analyses. We used QFT-GIT culture negative (nil) controls supernatants for analyses because serum samples were not available for all the individuals studied. We have previously shown no differences in antibody concentrations between QFT-GIT nil supernatants and serum [33].

### Ethical approvals

This study was approved by the Makerere University School of Medicine Research & Ethics Committee, the Uganda National Council for Science & Technology and the London School of Hygiene & Tropical Medicine (LSHTM) Research Ethics Committee. Written informed consent was obtained from all study participants before collection of samples. The study participants also consented to the use of their specimen for immunological investigations. All samples were only identified by unique laboratory numbers so as to maintain the study participants’ anonymity.

### Antigens

Recombinant protein, Ag85A (NR-14871) was obtained through BEI Resources, National Institute of Allergy and Infectious Diseases, National Institutes of Health. Human purified alpha-1 antitrypsin was obtained from MP Biomedicals, USA.

### Antibody dilution ELISA

Antibody avidity measurements using chaotrope-based dissociation assays can be influenced by differences in antibody amount [34]. We therefore measured anti-Ag85A antibody levels at several serial dilutions to estimate the sample specific dilution needed to achieve a similar antibody level across all the samples prior to the thiocyanate elution assays. In this procedure, Immunlon 4 HBX microtitre plates (Thermoscientific, USA) were coated overnight at 4°C with 50 μl/well Ag85A antigen diluted to a concentration of 5μg/ml in carbonate-bicarbonate coating buffer. The plate wells were then washed four times with phosphate buffered saline containing 0.05% Tween20 (PBST) and blocked with 150 μl/well blocking solution (1% skimmed milk in PBST) for 2 h at room temperature. Two-fold serial dilutions of samples were prepared starting with a dilution of 1/100 and ending at a dilution of 1/800 and then 50 μl incubated in plate wells overnight at 4°C. All samples were run in duplicate to reduce chances of error. The plates were washed as before and then 50 μl/well of anti-human IgG horseradish peroxidase conjugate (Dako, Denmark) was incubated in the plate wells for 1 h at room temperature. The plates were washed four times and then 100 μl of the substrate, 3,3’,5,5’-tetramethylbenzidine (BD Biosciences-USA) containing hydrogen peroxide, was incubated in each well for 15 minutes. The reaction was stopped by adding 25 μl of 2 M sulphuric acid to each well and then the optical density (OD) read in an ELISA reader (BioTek) at a test wavelength of 450 nm and a reference wavelength 630 nm. The difference between the two readings was computed to control for background optical interference and was taken as the final OD result. These values were then plotted against the sample dilutions and curves were generated using cubic spline regression models [35] as illustrated in Figure A in S1 Fig of the supporting information. The equations of these curves were then used to determine sample dilutions corresponding to a uniform OD value of 0.4. Antibody titres were obtained by getting the inverse of this dilution.

### Ammonium thiocyanate ELISA

Immunlon 4 HBX microtitre plate (Thermoscientific, USA) were coated and blocked as described above. Samples were individually diluted to give an approximately uniform OD as determined by antibody dilution ELISAs and 50 μl/well incubated on the plates at 4°C overnight. As with the previous assay, all samples were run in duplicate. The plates wells were then washed four times with PBST and then 50 μl/well ammonium thiocyanate (Sigma) at varying concentrations (0.0 to 1.0 M) was incubated in the plate wells for 10 min at room temperature. The plates were washed six times and then 50 μl/well of anti-human IgG horseradish peroxidase conjugate was incubated in the plate wells for an hour at room temperature. The plates were washed four times, developed with 3,3’,5,5’-tetramethylbenzidine substrate for 15 min and ODs read after the reaction was stopped with 2 M sulphuric acid. The OD readings in the presence of ammonium thiocyanate were then converted into the percentage of bound antibody in the absence of the reagent (0 M ammonium thiocyanate). These percentages were then plotted against corresponding concentrations of ammonium thiocyanate and curves fitted following a non-linear regression model. The equations of these curves were used to determine the concentration of ammonium thiocyanate required to reduce the OD of a particular sample by 50%. This value is referred to as the avidity index. A representative curve is shown in Figure B in S1 Fig.

### Surface plasmon resonance

#### Antigen immobilisation

Prior to coupling, Ag85A was dialysed against 2 mM HEPES containing 0.8% sodium chloride. The antigen was then immobilised onto CM5 sensor chips (Biacore, GE Healthcare, Amersham) by amine coupling using a Biacore 3000 instrument (Biacore, GE Healthcare, Amersham). Human purified alpha-1 antitrypsin was immobilised onto a separate flow cell of the chip as a control for non-specific binding. In the immobilisation procedure, chip surfaces were cleaned with a 10 μl volume of 50 mM sodium hydroxide. The carboxymethyl groups on these surfaces were then activated by injecting 40 μl of a mixture of 0.4 M 1-ethyl-3-(3-dimethylaminopropyl)-carbodiimide (EDC) and 0.1 M N-hydroxysuccinimide (NHS) in a ratio of 1:1 at a rate of 5 μl/min. pH scouting for antigen immobilisation revealed that the best pH for Ag85A immobilisation was 4.5 and 4.25 for alpha-1 antitrypsin. Antigen diluted to 20 μg/ml in 10 mM acetate buffer at these respective pHs was injected over the chip surface at a rate of 5 μl/min in order to achieve an increase in RU of approximately 2000 RU above background. Such immobilisation levels were sufficient to increase sensitivity without leading to bivalent interactions. After the pre-requisite amount of antigen was bound to the chip, the unused activated carboxymethyl groups were deactivated by injection of 40 μl 1 M ethanolamine (pH 8.5) at a rate of 5 μl/min. The chip was then ready for sample testing.

#### Sample preparation and analysis

Direct analysis of the QFT-GIT supernatants showed considerable background non-specific binding to surfaces particularly the control flow cell. In order to counter this the samples were pre-treated to remove low molecular weight components using polyacrylamide gel spin columns. This was shown to be effective at reducing this background binding and was adopted for all subsequent analyses. In this procedure, QFT-GIT supernatants were diluted 1 in 3 in HBSPE (10 mM HEPES pH 7.4 containing 0.15 M NaCl, 3 mM EDTA and 0.005% (w/v) Surfactant P20; GE Healthcare) running buffer and then passed through a Bio-Gel P-30 (Bio-Rad, UK) polyacrylamide gel spin column equilibrated in HBSPE by centrifugation at 1000 g for 4 min. The gel filtrate was harvested and further diluted 1 in 8 in HBSPE running buffer containing 1% carboxymethyl-dextran sodium salt (Sigma) in preparation for Biacore analysis.

Prepared samples were then analysed in the Biacore 3000 instrument at a temperature of 25°C. In this procedure, 90 μl of each sample was injected (kinject) over the chip surface at a rate of 15 μl/min (6 min) and then allowed to dissociate from the chip surface for 8 min. No mass transfer effects were seen under these conditions. In order to re-use the chip, the surface was regenerated by eluting any remaining bound antibody by injecting 10 μl of 50 mM HEPES containing 3 M MgCl_2_ and 25% ethylene glycol followed by 10 μl of 20 mM glycine pH 1.5 all at a rate of 15 μl/min. This was followed by an injection of 150 μl HBSPE running buffer at the same rate to wash away elution reagents before injection of the next sample. This procedure was followed after analysis of each sample.

The results produced were in the form of sensograms; curves showing changes in RU over time. BIAevaluation software version 4.1.1 (Biacore, GE Healthcare, Amersham) was used for analysis of these data. Background from the alpha-1 antitrypsin control flow cell was subtracted following curve alignment. Dissociation rate was determined using a 1:1 Langmuir model between 10 seconds and 300 seconds after the end of sample injection.

In order to further reduce non-specific binding, newly prepared chip surfaces were treated with fetal bovine serum (FBS; Sigma) prior to sample analysis. This was achieved by injecting at least 10, 90 μl volumes of FBS diluted 1 in 10 in HBSPE running buffer over the chip surface following the procedure described for sample analysis. A control sample was included in every sample run to ensure reproducibility of assay results and monitor chip performance.

### Statistical analysis

Results were analysed using Stata release statistical package (Statacorp LP, College Station, TX, USA) and GraphPad Prism software (GraphPad Inc., San Diego, CA). The initial analyses were made using the Wilcoxon rank-sum test to compare antibody measurements between uninfected controls, individuals with LTBI and active TB cases. After applying a Bonferroni correction for multiple testing only p values ≤ 0.017 were considered statistically significant during these comparisons.

Linear regression analysis with bootstrap confidence intervals was used to determine associations between *M*.*tb* infection status and antibody measurements [20,36]. In order to cater for multiple testing, we calculated q values for each of the comparisons between *M*.*tb* infection states and uninfected controls using the Bonferroni approach. Adjusting was done for the effects of potential confounders such as HIV infection, age, socioeconomic status (SES) and gender in the final analyses. Information on these exposures was available for almost all the study participants. As part of the parent study, infection with malaria, cytomegalovirus and helminths and the presence of a BCG scar was ascertained among the HHCs but due to the nature of the study, the same was not done in the APTB cases [32]. We therefore were not able to account for the effects of these various parasitic and viral coinfections on the antibody responses measured.

Further analyses involved the use of Spearman’s rank correlation to test for correlation between avidity results obtained by ammonium thiocyanate elution ELISAs and those obtained from SPR.

## Results

### Characteristics of study participants

Individuals included in the study were of varied age, gender, HIV serostatus and socio-economic status (SES) as shown in Table 1. The average age of APTB cases and individuals with LTBI was greater than those who were uninfected. Older age in the APTB cases was due to an inclusion criterion applied by the larger TB household contact study, favouring only adults over 18 years of age for recruitment to this group. Older age among those with LTBI was reported previously in this study group [20] and may reflect increased exposure to *M*.*tb* with age. As expected, the APTB group had a larger proportion of males [37], HIV seropositive individuals [38] and people of low socioeconomic status [39,40] compared to the other two groups.

**Table 1:** Characteristics of study participants.

Characteristic	Uninfected (n = 30)	LTBI (n = 34)	APTB (n = 75)	Total (n = 139)
**Mean age and range (years)**	15 (1, 66)	28 (1, 66)	30 (18, 50)	26 (1, 66)
**Males**	10 (33.3%)	10 (29.4%)	43 (57.3%)	63 (45.3%)
**HIV positive**	2 (6.7%)	1 (3.0%)	27 (36.0%)	30 (21.6%)
**Low SES ^a^**	12 (40%)	16 (47.1%)	44 (65.7%)	72 (55.0%)

LTBI: latent tuberculosis infection, APTB: active pulmonary tuberculosis, SES: socioeconomic status.

^a^ Individuals were either of low or medium socio-economic status.

### Antibody avidity for Ag85A as determined by chaotrope-based dissociation assays is greater in APTB cases

In our initial investigation, IgG antibody titres against Ag85A were measured using an antibody dilution ELISA in individuals of varied *M*.*tb* infection status, and thereafter their avidity determined using a thiocyanate-based chaotropic dissociation assay. This technique measures the amount of chaotropic reagent required to reduce antibody-antigen binding by 50%, which is referred to as the avidity index [28]. QFT-GIT nil control supernatants were used for analyses because we did not have serum samples for all the individuals studied. Due to restrictions in sample volumes, only 74 of the 139 individuals were investigated using the chaotrope-based dissociation assay, and these consisted of 9 uninfected controls, 11 individuals with LTBI and 54 APTB cases.

**Fig 1:**
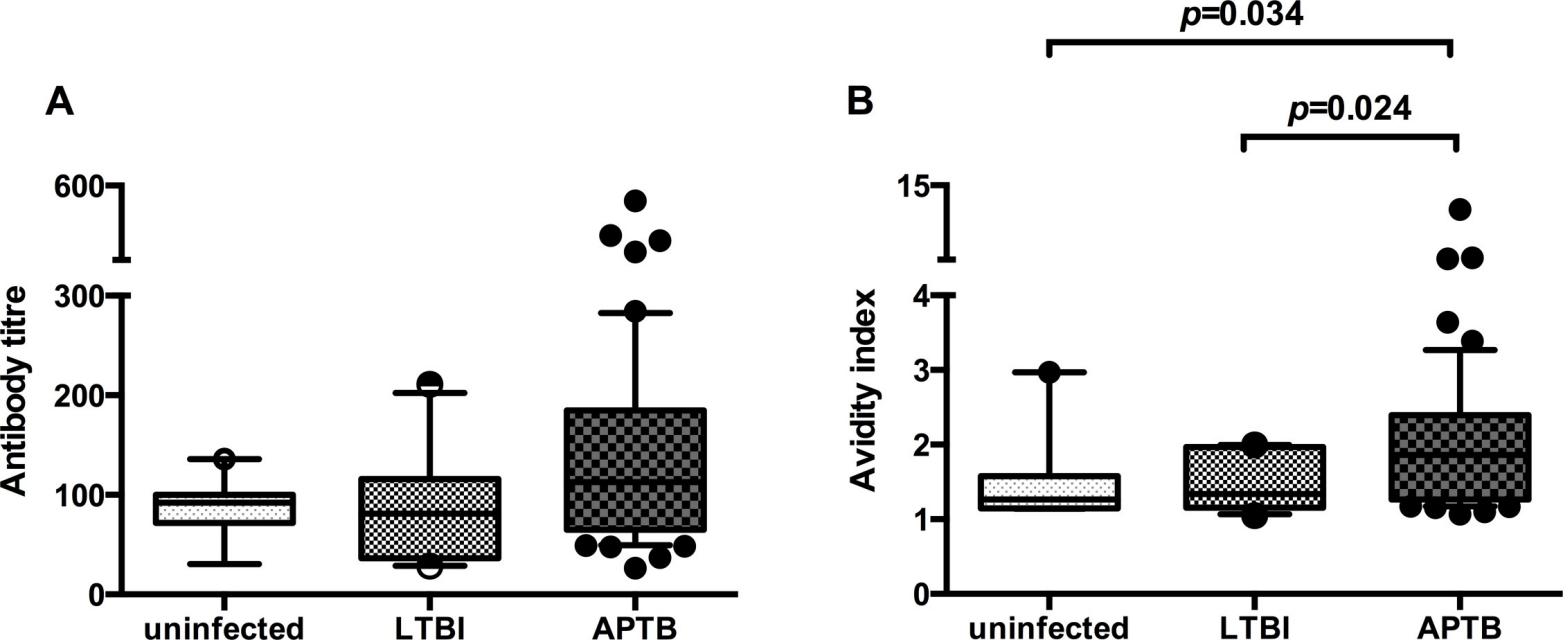
Differences in anti-Ag85A antibody avidity as measured by chaotrope-based dissociation assay between uninfected controls, individuals with latent TB infection (LTBI) and active pulmonary TB (APTB) cases. Panel A: antibody titres corresponding to the inverse of the sample dilution required to achieve an optical density (OD) of 0.4, Panel B: avidity index values from testing samples individually diluted to achieve a uniform OD of 0.4. The Wilcoxon rank-sum test was used to compare antibody titre and avidity between uninfected controls (n = 9), individuals with LTBI (n = 11) and APTB cases (n = 54). Whiskers represent the 10^th^ and 90^th^ percentile and dots represents samples outside the interquartile range. After correction for multiple testing using the Bonferroni approach, only *P* values ≤ 0.017 were considered statistical significant.

Initial preliminary analyses indicated that there were no statistically significant differences in anti-Ag85A antibody titre between the uninfected controls, individuals with LTBI and APTB cases but there was a trend towards higher antibody titres in the latter sub-group (Fig 1A). There was some evidence to suggest that the anti-Ag85A antibody avidity index was higher among the APTB cases in comparison to individuals with LTBI and the uninfected controls (Fig 1B), however, this was not statistically significant after correction for multiple testing. There were no differences in antibody avidity between the individuals with LTBI and uninfected controls.

Due to the differences in age, gender, HIV serostatus and socio-economic status (SES) between the uninfected controls, individuals with LTBI and APTB cases (Table 1) and their possible influence on immune responses, we compared antibody titres and avidity index values between our study sub-groups while adjusting for the effects of these potential confounders in our final analyses as shown in Table 2. There were higher titres of antibodies in APTB cases in comparison to the uninfected controls and individuals with LTBI. There were no differences between individuals with LTBI and the uninfected controls (Table 2). The adjusted analyses also indicated that the APTB cases had more avid antibodies in comparison to uninfected controls and individuals with LTBI (Table 3).

**Table 2:** Factors associated with anti-Ag85A antibody titre as measured by ELISA^b^.

Factor	Adjusted GMR (95%CI) ^c^	*P* value	*Q* value ^d^
**Age**	**1.000 (0.985–1.015)**	**0.965**	
**Gender**			
**Female**	1		
**Male**	1.025 (0.747–1.407)	0.877	
**HIV serostatus**			
**Negative**	1		
**Positive**	**0.571 (0.391–0.834)**	**0.004**	
**SES**			
**Low**	1		
**Medium**	1.162 (0.848–1.592)	0.351	
***M*.*tb* infection state**			
**Uninfected**	1		
**LTBI**	0.941 (0.548–1.615)	0.825	1.000
**APTB**	**1.758 (1.139–2.714)**	**0.011**	**0.033**
**APTB Vs LTBI^§^**	**1.869 (1.150–3.037)**	**0.012**	**0.036**

GMR: geometric mean ratio, LTBI: latent tuberculosis infection, APTB: active pulmonary tuberculosis, SES: socioeconomic status.

^b^ 9 uninfected controls, 11 LTBI and 48 APTB cases.

^c^ Results were generated using linear regression and the factors were adjusted for one another.

^d^ Q values were computed for multiple comparisons between *M*.*tb* infection states and uninfected controls using the Bonferroni method.

^§^ LTBI is baseline comparison group.

Crude GMRs can be found in S1 Table in the supporting information.

**Table 3:** Factors associated with chaotrope-based anti-Ag85A antibody avidity ^e^.

Factor	Adjusted GMR (95%CI) ^f^	*P* value	*Q* value ^g^
**Age**	1.000 (0.993–1.007)	0.978	
**Gender**			
**Female**	1		
**Male**	0.861 (0.722–1.027)	0.098	
**HIV serostatus**			
**Negative**	1		
**Positive**	**0.677 (0.567–0.812)**	**<0.001**	
**SES**			
**Low**	1		
**Medium**	**1.317 (1.052–1.649)**	**0.016**	
***M*.*tb* infection state**			
**Uninfected**	1		
**LTBI**	1.024 (0.738–1.419)	0.889	1.000
**APTB**	**1.641 (1.153–2.337)**	**0.006**	**0.018**
**APTB Vs LTBI^†^**	**1.604 (1.282–2.006)**	**<0.001**	**<0.001**

GMR: geometric mean ratio, LTBI: latent tuberculosis infection, APTB: active pulmonary tuberculosis, SES: socioeconomic status.

^e^ 9 uninfected controls, 11 LTBI and 48 APTB cases.

^f^ Results were generated using linear regression and the factors were adjusted for one another.

^g^ Q values were computed for multiple comparisons between *M*.*tb* infection states and uninfected controls using the Bonferroni method.

^†^ LTBI is baseline comparison group.

Crude GMRs can be found in S2 Table in the supporting information.

### HIV seropositivity and socioeconomic class are associated with chaotrope-based anti-Ag85A antibody avidity

Our adjusted analyses showed that HIV seropositivity was associated with a decrease in antibody titre (Table 2). This observation is in agreement with previous findings and was largely expected [20]. HIV seropositivity was associated with a lower anti-Ag85A antibody avidity index while medium SES was associated with a higher antibody avidity index in comparison to low SES (Table 3). There was also weak evidence suggesting that male gender was associated with less avid antibodies. We found no associations between age and antibody avidity determined by chaotrope-based dissociation assays.

### SPR determined anti-Ag85A antibody avidity is higher in *M*.*tb* infected individuals

Next, we evaluated the avidity of antibodies against Ag85A based upon dissociation rates. These were determined by analysing QFT-GIT nil control supernatants by SPR using the Biacore biosensor platform.

Only 88 of the 139 individuals included in the study were assessed using SPR because of limited sample volumes. These included 25 uninfected controls, 23 individuals with LTBI and 40 APTB cases. Two representative SPR traces are shown in the supporting information, one from a sample with relatively low anti-Ag85A antibody RU and a fast dissociation rate from the uninfected sub-group and one with a relatively high anti-Ag85A antibody RU and a slow dissociation rate from the APTB sub-group (S2 Fig). These values were compared between the uninfected controls, individuals with LTBI and APTB cases in the preliminary analyses.

**Fig 2:**
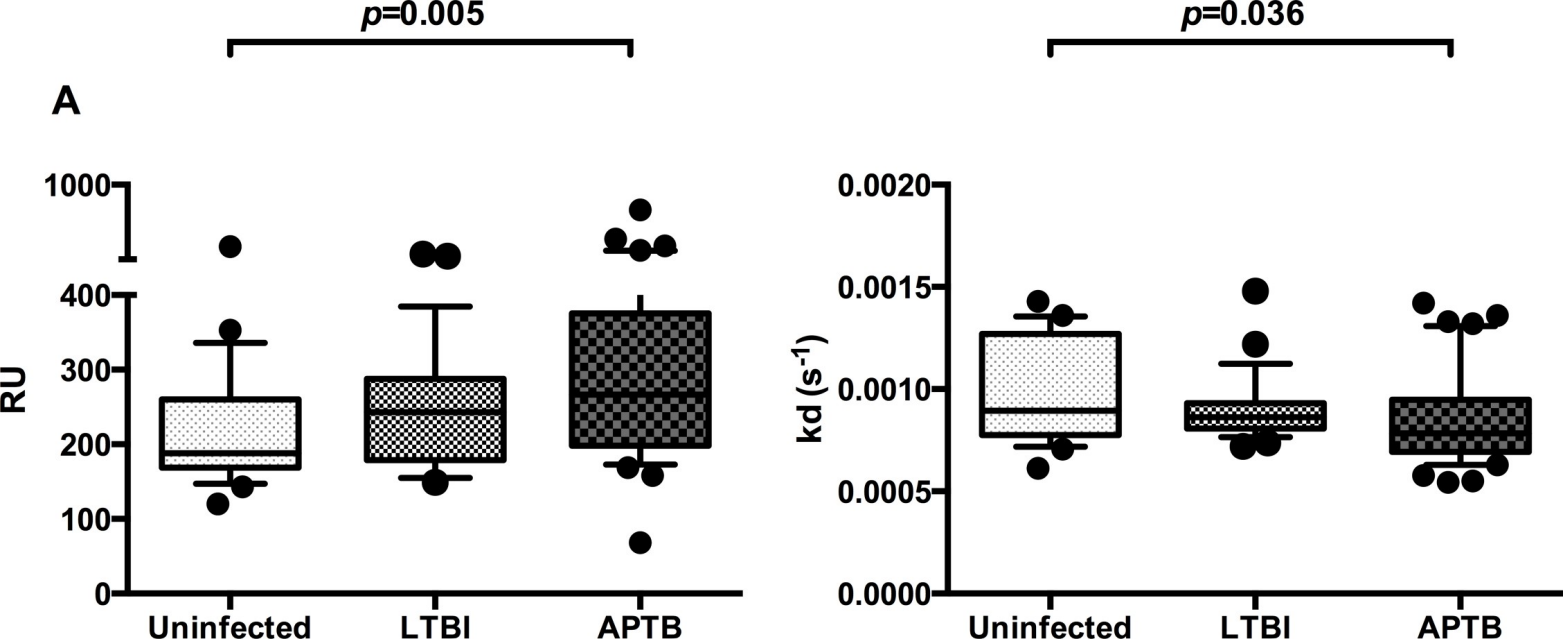
Differences in anti-Ag85A antibody responses and dissociation rates between uninfected controls, individuals with latent TB infection (LTBI) and active pulmonary TB (APTB) cases. Panel A: response units (RU), Panel B: dissociation rates [kd(s^-1^)]. The Wilcoxon rank-sum test was used to compare antibody responses and dissociation rates between uninfected controls (n = 23), individuals with LTBI (n = 25) and APTB cases (n = 40). Whiskers represent the 10^th^ and 90^th^ percentile and dots represents samples outside the interquartile range. After correction for multiple testing using the Bonferroni approach, only *P* values ≤ 0.017 were considered statistical significant.

There was a higher anti-Ag85A antibody RU in the APTB cases compared to the uninfected controls (Fig 2A). In addition, anti-Ag85A antibodies in APTB cases had a slower dissociation rate, an indication of higher avidity, compared to those in the uninfected controls (Fig 2B) but this was not statistically significant after adjusting for multiple testing.

As with the avidity index data from the chaotrope-based assays, we compared RU and dissociation rates between our study sub-groups while adjusting for the effects of age, gender, HIV serostatus and SES in our final analyses. The differences observed in the initial preliminary analyses remained with APTB cases having higher anti-Ag85A antibody RU and slower dissociation rates compared to the uninfected controls (Table 4 and Table 5 respectively). Adjusted analysis of SPR data showed there were no differences in anti-Ag85A antibody RU between APTB cases and individuals with LTBI, and no differences in dissociation rate between the two. Additionally, it revealed evidence of higher anti-Ag85A antibody RU and slower dissociation rates in individuals with LTBI compared to the uninfected controls, however, this was not statistically significant after adjusting for multiple testing.

**Table 4:** Factors associated with anti-Ag85A antibody responses determined by SPR ^h^.

Factor	Adjusted GMR (95%CI) ^i^	*P* value	*Q* value ^j^
**Age**	0.996 (0.990–1.001)	0.128	
**Gender**			
**Female**	1		
**Male**	1.004 (0.844–1.195)	0.962	
**HIV serostatus**			
**Negative**	1		
**Positive**	0.983 (0.764–1.263)	0.892	
**SES**			
**Low**	1		
**Medium**	1.076 (0.908–1.275)	0.398	
***M*.*tb* infection state**			
**Uninfected**	1		
**LTBI**	1.213 (1.007–1.459)	0.041	0.123
**APTB**	**1.446 (1.151–1.817)**	**0.002**	**0.006**
**APTB Vs LTBI^¢^**	1.192 (0.970–1.466)	0.095	0.285

GMR: geometric mean ratio, LTBI: latent tuberculosis infection, APTB: active pulmonary tuberculosis, SES: socioeconomic status.

^h^ 23 uninfected controls, 24 LTBI and 34 APTB cases.

^i^ Results were generated using linear regression and the factors were adjusted for one another.

^**j**^ Q values were computed for multiple comparisons between *M*.*tb* infection states and uninfected controls using the Bonferroni method.

^¢^ LTBI is baseline comparison group.

Crude GMRs can be found in S3 Table in the supporting information.

**Table 5:** Factors associated with anti-Ag85A antibody dissociation rates ^k^.

Factor	Adjusted GMR (95%CI) ^m^	*P* value	*Q* value ^n^
**Age**	1.003 (1.000–1.007)	0.051	
**Gender**			
**Female**	1		
**Male**	1.051 (0.936–1.180)	0.400	
**HIV serostatus**			
**Negative**	1		
**Positive**	0.979 (0.832–1.152)	0.799	
**SES**			
**Low**	1		
**Medium**	0.937 (0.840–1.045)	0.242	
***M*.*tb* infection state**			
**Uninfected**	1		
**LTBI**	0.871 (0.763–0.994)	0.041	0.123
**APTB**	**0.796 (0.681–0.932)**	**0.004**	**0.012**
**APTB Vs LTBI^¶^**	0.915 (0.809–1.034)	0.152	0.456

GMR: geometric mean ratio, LTBI: latent tuberculosis infection, APTB: active pulmonary tuberculosis, SES: socioeconomic status.

^k^ 23 uninfected controls, 24 LTBI and 34 APTB cases.

^m^ Results were generated using linear regression and the factors were adjusted for one another

^n^ Q values were computed for multiple comparisons between *M*.*tb* infection states and uninfected controls using the Bonferroni method.

^¶^ LTBI is baseline comparison group.

Crude GMRs can be found in S4 Table in the supporting information.

We found no associations between age, gender, HIV serostatus and SES and anti-Ag85A antibody RU in our adjusted analyses (Table 5). We observed that older age was associated with a slightly faster dissociation rate but the evidence for this was weak (Table 5). Gender, HIV serostatus and SES were not associated with dissociation rate.

### Poor correlation between chaotrope and SPR determined avidity

**Fig 3:**
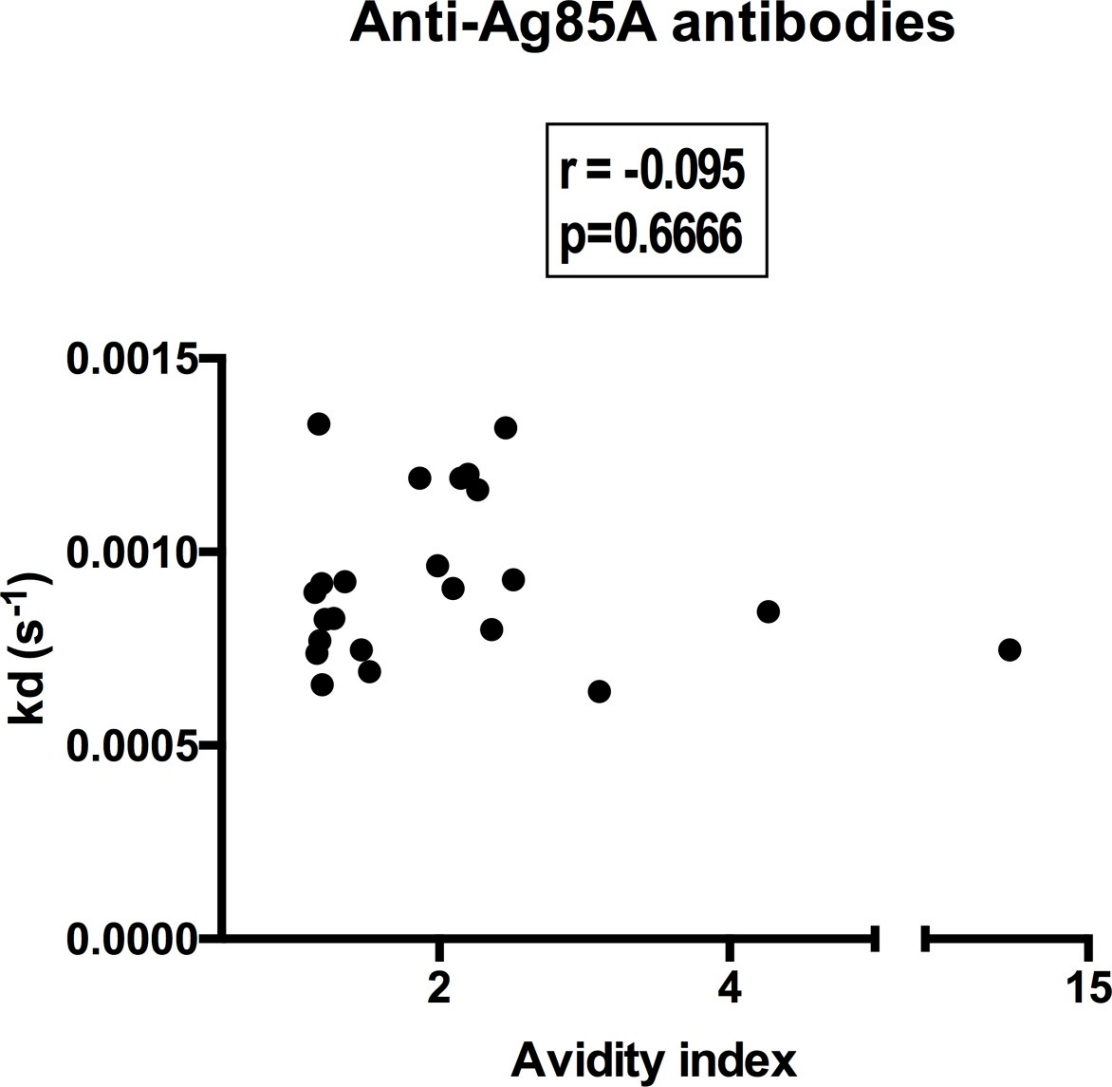
Anti-Ag85A antibody dissociation rates from SPR do not correlate with chaotrope-based avidity index values. Correlation coefficient and *p*-value were determined using Spearman's rank correlation. N = 23 (2 uninfected controls, 2 LTBI and 19 APTB cases).

In order to determine how well chaotrope and SPR-based measures of avidity compared with one another, we investigated the correlation between the two tests using data from 23 individuals who were analysed using both of these techniques. These comprised, 2 uninfected controls, 2 individuals with LTBI and 19 APTB cases. We found no correlation between anti-Ag85A antibody dissociation rates from the SPR and anti-Ag85A antibody avidity index values from the chaotrope-based assays (Fig 3).

## Discussion and conclusion

To our knowledge, this is the first study to characterise the avidity of *Mycobacterium* specific antibodies in individuals of varied *M*.*tb* infection status using SPR derived dissociation rates. We investigated the avidity of antibodies against Ag85A, an immunodominant *M*.*tb* antigen and a major constituent of several new anti-TB vaccine candidates [31]. We showed that APTB was associated with the presence of anti-Ag85A antibodies of higher avidity compared to those found in the uninfected controls. This observation agreed with findings from the more established chaotrope-based dissociation assays [28]. SPR analyses also revealed marginal evidence of higher antibody avidity among individuals with LTBI in comparison to uninfected controls. Our findings suggest that there is increased affinity maturation during *M*.*tb* infection. It remains to be seen whether increases in antibody avidity impact on antibody mediated control of bacillary growth and/or protection from infection.

Our results agree with findings from Arias-Bouda *et al*. [28], who reported that there were more avid antibodies against mycobacterial cell lysates in APTB cases compared to controls, as assessed by chaotrope-based assays. Perley *et al*. also reported a similar increase in antibody avidity among APTB cases when mycobacterial antigen fractions (although not individual antigens) were used for chaotrope-based measurement of binding strength [27]. These findings, together with our own, show that there may be significant affinity maturation in individuals with pulmonary TB. There is also evidence that active TB disease activates *Mycobacterium* specific memory B cells generated from past exposure [41]. These B cells would have already undergone affinity maturation in the B cell follicles and could contribute to the high affinity antibodies observed. Indeed, Zimmermann *et al*. reported increased somatic hypermutation in plasmablast populations from APTB cases, and suggested that these cells may have originated from memory B cells [19]. It is important, however, to mention that the APTB cases included in our study comprised a large number of individuals who had begun anti-mycobacterial drug therapy. Treatment has been reported to affect avidity of anti-mycobacterial antibodies [28] and may therefore affect our findings.

Our study characterised antibody avidity in individuals with defined LTBI status and found marginal evidence of increased affinity maturation in these individuals in our SPR analyses. TB latency is often thought to be associated with low numbers of mycobacteria that are largely metabolically inactive. However, the current understanding is that this state is actually heterogeneous and is composed of individuals with granulomas at different stages of breakdown and reformation [42]. Further investigations involving larger numbers of individuals may improve upon the power to detect significant changes in antibody avidity as a result of LTBI.

Some have proposed the use of antibody avidity assays for TB diagnosis [28], however, we observed high levels of variation in anti-Ag85A antibody avidity within the uninfected control, individuals with LTBI and APTB cases and a lot of overlap between these three groups. This may limit the use of chaotrope-based dissociation assays and SPR in the detection of TB in this population. The presence of anti-Ag85A antibodies of appreciable avidity in the uninfected group may be related to prior BCG vaccination and/or exposure to *M*.*tb* or non-tuberculous mycobacteria, although this remains to be proven. Exposure to *M*.*tb* is also highly likely because these individuals were household contacts of APTB cases and are from Uganda, a country with a high TB burden [1].

In addition to our main findings, we found that HIV seropositivity was associated with lower anti-Ag85A antibody titre and avidity while medium SES was associated with higher anti-Ag85A antibody avidity from chaotrope-based assays. HIV proteins have been shown to reduce activation of B cells following antigen stimulation and hamper their ability to proliferate [43]. Infection with HIV also alters follicular T helper cell function which may negatively impact the affinity maturation process giving rise to antibodies of low avidity [44]. Our observations agree with a study by Nair and colleagues which described reduced quantity and avidity of measles virus specific antibodies in HIV infected children [45]. We are unsure as to why individuals of medium SES had antibodies of a higher avidity index especially after considering the fact that there were fewer APTB cases (32.7%) and individuals with LTBI (45.5%) in the medium SES group in comparison to the low SES group. We hypothesise that factors other than *M*.*tb* infection, such as prior BCG vaccination, may account for this difference.

The differences between the results from SPR and chaotrope-based dissociation assays may reflect differences in the sensitivities of these assays. Chaotrope-based assays preferentially detect antibodies of high avidity, as these antibodies remain bound after dissociation with chaotropic reagents [34]. This limits the ability of these techniques to detect antibodies of intermediate or weak binding strength. SPR, on the other hand, is able to measure the avidity of such antibodies shortly after the end of the association phase, and this improves its sensitivity because it can detect weakly interacting antibody with a relatively fast dissociation-rate. The chaotrope-based assays also preferentially disrupt conformational epitopes whilst more linear epitopes are relatively resistant [46], with no such bias seen in SPR analyses. This may negatively impact on the sensitivity of the chaotrope-based assay for the measurement of avidity of antibodies against non-linear epitopes. It is also important to understand that unlike the chaotrope-based assay, the SPR assay measures binding regardless of antibody isotype and therefore includes data from non-IgG antibodies. Although IgG makes up the bulk of antibody in these samples, this may contribute to the differences in the results from these two assays.

We were not able to test all individuals using both SPR and chaotrope-based assays due to limitations in sample volumes. The differences in some of the findings from SPR and chaotrope-based assays may therefore also reflect variations in the antibody responses in our study participant population. However, when we determined the association between SPR and chaotrope-based avidity data from individuals tested using both techniques, we found that they did not correlate with one another. This is in agreement with findings from Reddy and others who described avidity of antibodies against the *Plasmodium falciparum* antigens Apical Membrane Protein-1 and Merozoite Surface Protein-2 [29]. This study was also not able to find any correlation between SPR determined dissociation rate and chaotrope-based avidity index values amongst the individuals investigated. This observation, as well as our own, puts forward the argument that the differences in the results of SPR and chaotrope-based assays are due to fundamental differences in these techniques.

The SPR and chaotrope-based assays measure different aspects of antibody avidity, with the former characterising the kinetics of antibody-antigen interactions in relation to time and the latter describing resistance of antibody-antigen binding to disruption by chaotropic reagents. Our dissociation rate measurements supplement the findings from chaotrope-based dissociation assays because they provide an indication of the ability of an antibody to remain bound for longer periods. This attribute may facilitate antibody engagement in effector functions with other immune cells [29]. Indeed, a recent study by Botterman and colleagues has shown that slow dissociation rates may be necessary to facilitate antibody Fc-Fc receptor signalling [30]. Studies of influenza (H5N1) vaccine responses have also revealed that the serum titre necessary for viral neutralisation is much higher for anti-haemagglutinin antibodies of slower dissociation rates than those with fast dissociation rates [47].

We chose Ag85A antigen for our study because of its immunogenicity, its importance as a constituent of several vaccine candidates [31] and the fact that antibody responses towards it have been associated with protection from TB in humans [7]. Our findings are therefore important to the TB vaccine field because they show that avid antibodies are generated against a principle vaccine antigen in human subjects. Although anti-Ag85A antibody avidity was raised in APTB cases beyond the levels observed in the uninfected controls using both avidity index and dissociate rate measures, we are unaware of the impact of the size of these changes on antibody-mediated control of mycobacterial infection. It would therefore be important in future studies to determine whether the magnitude of increase in antibody avidity affects more functional immunological measures of anti-TB immunity such as the *in vitro* mycobacterial growth inhibition [48]. It would also be important to determine whether BCG vaccination induces the production of high avidity anti-Ag85A antibodies and whether they have any role in protection against *M*.*tb* infection and disease.

We used QFT-GIT culture nil supernatants for our investigations. It is possible that antibodies secreted during the period of culture may have different glycosylation properties [49] and this could potentially affect antibody binding and hence our results [50]. However, we have previously shown a lack of difference in antibody concentration between QFT-GIT nil supernatants and serum [33]. This implies that the secretion of new antibodies into whole blood over the 16–24 hour QFT-GIT culture period is not sufficient to impact on antibody levels and hence may have minimal effects on our findings.

In conclusion, our study shows that antibodies of increased avidity are produced in *M*.*tb* infected individuals. More research is needed to determine whether they have any contribution towards antibody-mediated control of infection.

## Supporting information

S1 Fig Representative curves from antibody dilution and ammonium thiocyanate ELISAs. Panel A: anti-Ag85A optical density levels at increasing sample dilutions. Curves were fitted using cubic spline regression models. Panel B: percentages of bound antibody at increasing molar concentrations of ammonium thiocyanate. Curves were fitted using non-linear regression models.
(TIFF)

S2 Fig Sensogram from SPR analysis of two representative samples. Association and dissociation phases are shown for anti-Ag85A antibodies following subtraction of alpha-1 antitrypsin background control responses. Sample A is from the active pulmonary TB group and has a high anti-Ag85A RU and relatively slow dissociation rate while sample B is from the uninfected control group and has a low anti-Ag85A RU and relatively fast dissociation rate. Arrows indicate the point at which RU was measured for the respective curves. Slopes were fitted using a 1:1 Langmuir model and were used to approximate dissociation rate [kd (s^-1^)].
(TIFF)

S1 Table Factors associated with anti-Ag85A antibody titre as measured by ELISA before adjusting for the effects of confounders. (DOCX)

S2 Table Factors associated with chaotrope-based anti-Ag85A antibody avidity before adjusting for the effects of confounders. (DOCX)

S3 Table Factors associated with anti-Ag85A antibody responses determined by SPR before adjusting for the effects of confounders. (DOCX)

S4 Table Factors associated with anti-Ag85A antibody dissociation rates before adjusting for the effects of confounders. (DOCX)

S1 File Dataset 1.0. (XLSX)
