# Supplementary figures and images for: Characterising antibody avidity in individuals of varied Mycobacterium tuberculosis infection status using surface plasmon resonance
Source: PLoS One. 2018 Oct 12;13(10):e0205102. doi: 10.1371/journal.pone.0205102 (PMC6185725; doi:10.1371/journal.pone.0205102)

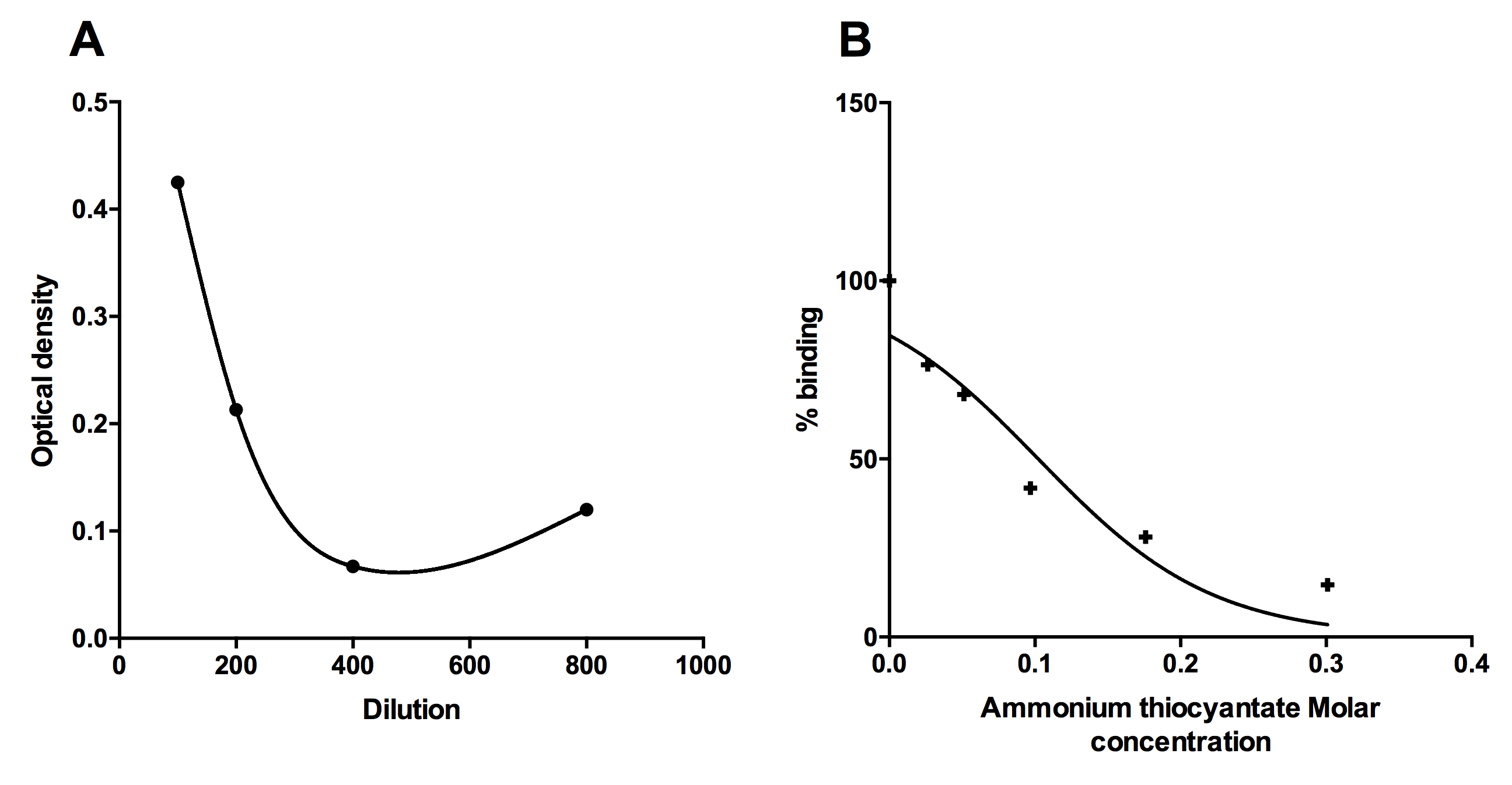

Supplement: S1 Fig — Panel A: anti-Ag85A optical density levels at increasing sample dilutions. Curves were fitted using cubic spline regression models. Panel B: percentages of bound antibody at increasing molar concentrations of ammonium thiocyanate. Curves were fitted using non-linear regression models. (TIFF) [file pone.0205102.s001.tiff]

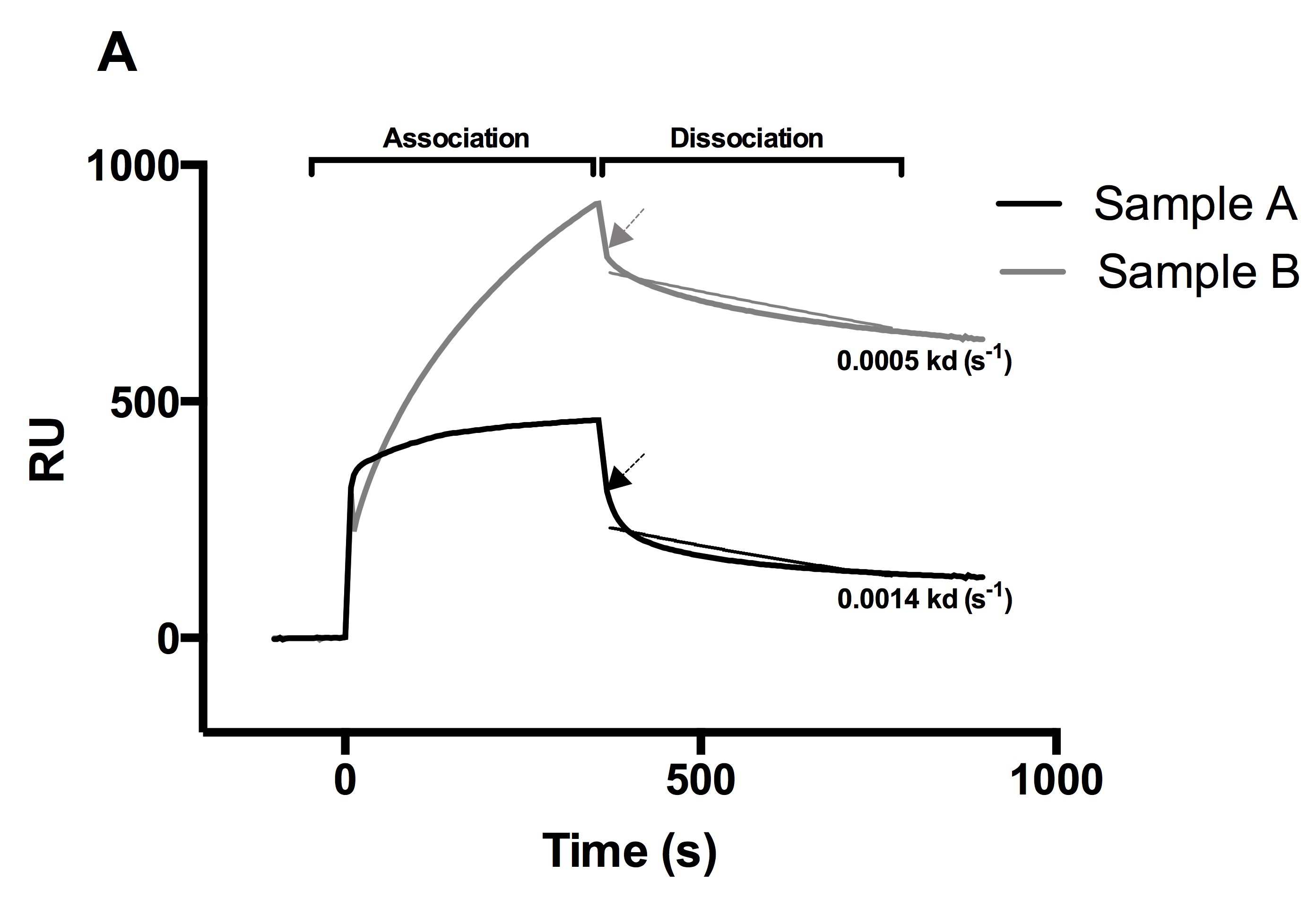

Supplement: S2 Fig — Association and dissociation phases are shown for anti-Ag85A antibodies following subtraction of alpha-1 antitrypsin background control responses. Sample A is from the active pulmonary TB group and has a high anti-Ag85A RU and relatively slow dissociation rate while sample B is from the uninfected control group and has a low anti-Ag85A RU and relatively fast dissociation rate. Arrows indicate the point at which RU was measured for the respective curves. Slopes were fitted using a 1:1 Langmuir model and were used to approximate dissociation rate [kd (s-1)]. (TIFF) [file pone.0205102.s002.tiff]
