# Supplementary material for: Characterising antibody avidity in individuals of varied Mycobacterium tuberculosis infection status using surface plasmon resonance
Source: PLoS One. 2018 Oct 12;13(10):e0205102. doi: 10.1371/journal.pone.0205102 (PMC6185725; doi:10.1371/journal.pone.0205102)
Supplement: S1 Table — (DOCX) [file pone.0205102.s003.docx]

**S1 Table.**

| Factor ^a^ | Crude GMR (95%CI) | *P* value | *Q* value ^b^ |
| --- | --- | --- | --- |
| Age | **1.004 (0.990-1.018)** | **0.566** |  |
| Gender |  |  |  |
| Female | 1 |  |  |
| Male | 1.147 (0.834-1.577) | 0.399 |  |
| HIV serostatus |  |  |  |
| Negative | 1 |  |  |
| Positive | **0.699 (0.493-0.991)** | **0.044** |  |
| SES |  |  |  |
| Low | 1 |  |  |
| Medium | 1.145 (0.834-1.571) | 0.402 |  |
| *M.tb* infection state |  |  |  |
| Uninfected | 1 |  |  |
| LTBI | 0.925 (0.577-1.485) | 0.749 | 1.000 |
| APTB | 1.419 (1.028-1.959) | 0.033 | 0.099 |
| APTB Vs LTBI^§^ | 1.533 (0.982-2.394) | 0.060 | 0.180 |

GMR: geometric mean ratio, LTBI: latent tuberculosis infection, APTB: active pulmonary tuberculosis, SES: socioeconomic status

^a^ 9 uninfected controls, 11 LTBI and 48 APTB cases

^b^ Q values computed for multiple comparisons between *M.tb* infection states and uninfected controls

^§^ LTBI is baseline comparison group
