# Supplementary material for: Characterising antibody avidity in individuals of varied Mycobacterium tuberculosis infection status using surface plasmon resonance
Source: PLoS One. 2018 Oct 12;13(10):e0205102. doi: 10.1371/journal.pone.0205102 (PMC6185725; doi:10.1371/journal.pone.0205102)
Supplement: S2 Table — (DOCX) [file pone.0205102.s004.docx]

**S2 Table.**

| Factor ^d^ | Crude GMR (95%CI) | *P* value | *Q* value ^e^ | |
| --- | --- | --- | --- | --- |
| Age | 1.003 (0.995-1.010) | 0.459 |  |  |
| Gender |  |  |  |  |
| Female | 1 |  |  |  |
| Male | 0.916 (0.751-1.117) | 0.388 |  |  |
| HIV serostatus |  |  |  |  |
| Negative | 1 |  |  |  |
| Positive | **0.800 (0.660-0.970)** | **0.023** |  |  |
| SES |  |  |  |  |
| Low | 1 |  |  |  |
| Medium | **1.314 (1.059-1.630)** | **0.013** |  |  |
| *M.tb* infection state |  |  |  |  |
| Uninfected | 1 |  |  |  |
| LTBI | 1.011 (0.795-1.285) | 0.929 | 1.000 |  |
| APTB | **1.334 (1.072-1.660)** | **0.01** | **0.030** |  |
| APTB Vs LTBI^†^ | **1.319 (1.099-1.584)** | **0.003** | **0.009** |  |

GMR: geometric mean ratio, LTBI: latent tuberculosis infection, APTB: active pulmonary tuberculosis, SES: socioeconomic status

^d^ 9 uninfected controls, 11 LTBI and 48 APTB cases

^e^ Q values computed for multiple comparisons between *M.tb* infection states and uninfected controls

^†^ LTBI is baseline comparison group
