# Supplementary material for: Characterising antibody avidity in individuals of varied Mycobacterium tuberculosis infection status using surface plasmon resonance
Source: PLoS One. 2018 Oct 12;13(10):e0205102. doi: 10.1371/journal.pone.0205102 (PMC6185725; doi:10.1371/journal.pone.0205102)
Supplement: S3 Table — (DOCX) [file pone.0205102.s005.docx]

**S3 Table.**

| Factor ^e^ | Crude GMR (95%CI) | *P* value | *Q* value ^f^ | |
| --- | --- | --- | --- | --- |
| Age | 1.000 (0.995-1.005) | 0.985 |  |  |
| Gender |  |  |  |  |
| Female | 1 |  |  |  |
| Male | 1.080 (0.904-1.291) | 0.395 |  |  |
| HIV serostatus |  |  |  |  |
| Negative | 1 |  |  |  |
| Positive | 1.057 (0.873-1.278) | 0.571 |  |  |
| SES |  |  |  |  |
| Low | 1 |  |  |  |
| Medium | 0.994 (0.844-1.171) | 0.946 |  |  |
| Anti-Ag85A antibody response (RU) |  |  |  |  |
| Uninfected | 1 |  |  |  |
| LTBI | 1.129 (0.944-1.350) | 0.184 | 0.552 |  |
| APTB | **1.321 (1.100-1.586)** | **0.003** | **0.009** |  |
| APTB Vs LTBI^¢^ | 1.170 (0.977-1.401) | 0.087 | 0.261 |  |

GMR: geometric mean ratio, LTBI: latent tuberculosis infection, APTB: active pulmonary tuberculosis, SES: socioeconomic status

^e^ 23 uninfected controls, 24 LTBI and 34 APTB cases

^f^ Q values computed for multiple comparisons between *M.tb* infection states and uninfected controls

^¢^ LTBI is baseline comparison group
