# Supplementary material for: Characterising antibody avidity in individuals of varied Mycobacterium tuberculosis infection status using surface plasmon resonance
Source: PLoS One. 2018 Oct 12;13(10):e0205102. doi: 10.1371/journal.pone.0205102 (PMC6185725; doi:10.1371/journal.pone.0205102)
Supplement: S4 Table — (DOCX) [file pone.0205102.s006.docx]

**S4 Table.**

| Factor ^g^ | Crude GMR (95%CI) | *P* value | *Q* value ^h^ | |
| --- | --- | --- | --- | --- |
| Age | 1.000 (0.997-1.004) | 0.748 |  |  |
| Gender |  |  |  |  |
| Female | 1 |  |  |  |
| Male | 1.004 (0.898-1.124) | 0.940 |  |  |
| HIV serostatus |  |  |  |  |
| Negative | 1 |  |  |  |
| Positive | 0.962 (0.836-1.107) | 0.588 |  |  |
| SES |  |  |  |  |
| Low | 1 |  |  |  |
| Medium | 0.986 (0.887-1.095) | 0.787 |  |  |
| *M.tb* infection state |  |  |  |  |
| Uninfected | 1 |  |  |  |
| LTBI | 0.923 (0.817-1.043) | 0.199 | 0.597 |  |
| APTB | **0.864 (0.756-0.987)** | **0.031** | **0.093** |  |
| APTB Vs LTBI^¶^ | 0.936 (0.841-1.040) | 0.218 | 0.654 |  |

GMR: geometric mean ratio, LTBI: latent tuberculosis infection, APTB: active pulmonary tuberculosis, SES: socioeconomic status

^g^ 23 uninfected controls, 24 LTBI and 34 APTB cases

^h^ Q values computed for multiple comparisons between *M.tb* infection states and uninfected controls

^¶^ LTBI is baseline comparison group
